# A case of eosinophilic pneumonia simultaneously diagnosed in a patient and a tame cat: a case report

**DOI:** 10.1186/1752-1947-8-83

**Published:** 2014-03-04

**Authors:** Takao Tsuji, Mitsuko Kondo, Ryota Kikuchi, Etsuko Tagaya, Jun Tamaoki

**Affiliations:** 1 Department of Respiratory Medicine, Tokyo Medical University Ibaraki Medical Center, 3-20-1 Chuou, Ami, Inashiki, Ibaraki 300-0395, Japan; 2 First Department of Medicine, Tokyo Women’s Medical University School of Medicine, 8-1 Kawada-cho, Shinjuku-ku, Tokyo 162-8666, Japan

**Keywords:** Cat, Eosinophilic pneumonia, Inhaled environmental antigens

## Abstract

**Introduction:**

Chronic eosinophilic pneumonia is an idiopathic disorder of unknown etiology. Corticosteroid treatment provides a good response but recurrence frequently occurs after tapering of corticosteroid. Chronic eosinophilic pneumonia occurs predominantly in middle-aged women and non-cigarette smokers, which leads to the speculation that environmental antigens, particularly in the home, contribute to the etiology.

**Case presentation:**

A 66-year-old Japanese woman was given a diagnosis of chronic eosinophilic pneumonia for 8 years and was treated with prednisone. She developed respiratory symptoms again with tapering of prednisone (10mg/day). A chest radiograph revealed patchy shadows in her bilateral upper lung fields, and bronchoalveolar lavage fluid revealed marked eosinophilia. Based on negative findings for other causes of eosinophilia, the diagnosis of the recurrence of chronic eosinophilic pneumonia was established. She was treated with prednisone (20mg/day), which demonstrated rapid improvement. Around the same time, her tame cat developed oral breathing, tachypnea and peripheral eosinophilia. Chest radiography of the cat revealed ground-glass opacity in its bilateral upper lung fields. Eosinophilic pneumonia was also diagnosed in the cat that was treated by prednisone (3mg/day). Since eosinophilic pneumonia was diagnosed simultaneously in the patient and her tame cat, it can be suggested that inhaled environmental antigens in the home caused the eosinophilic pneumonia. After moving out of her home, she and the cat had no recurrence of eosinophilic pneumonia.

**Conclusions:**

Although chronic eosinophilic pneumonia is an idiopathic disorder of unknown etiology, our case suggests that inhaled environmental antigens in the home may be associated with the causes of chronic eosinophilic pneumonia. A pet’s disease may give us an important clue for the therapeutic approach of the owner’s disease.

## Introduction

Chronic eosinophilic pneumonia (CEP) is an idiopathic disorder of unknown etiology associated with an abnormal accumulation of eosinophils in the lung. Corticosteroid treatment provides a good response to clinical and radiological findings of CEP, but recurrence frequently occurs after tapering of corticosteroid. We report an unusual case of CEP whereby a patient and her tame cat were simultaneously diagnosed with eosinophilic pneumonia.

## Case presentation

**Figure 1:**
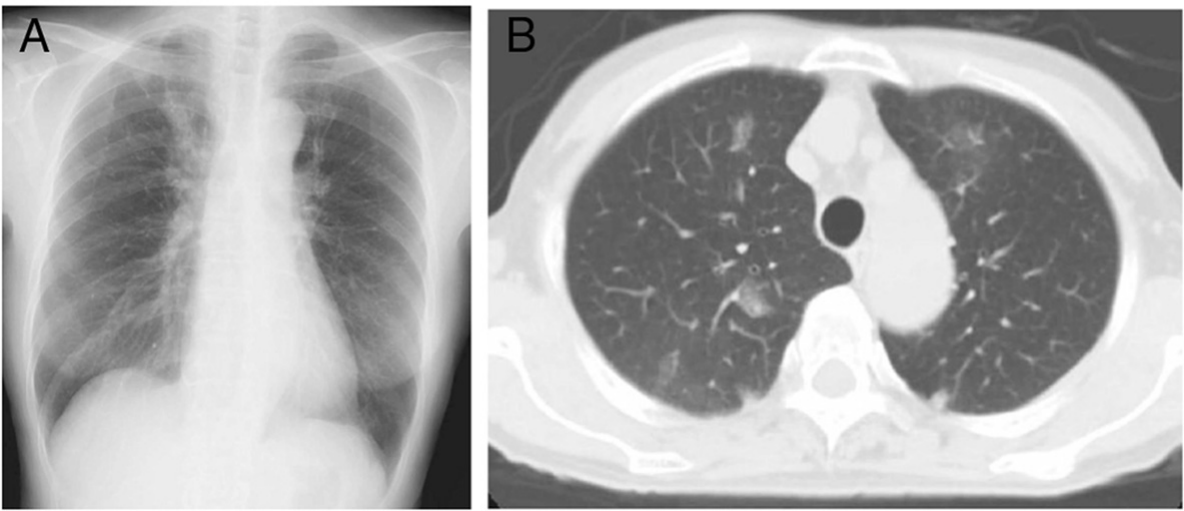
**Chest radiograph and chest computed tomography before prednisone treatment of 20mg/day. (A)** Chest radiograph revealing patchy shadows in the bilateral upper lung fields. **(B)** Chest computed tomography showing bilateral patchy ground-glass opacities with subpleural predominance.

**Figure 2:**
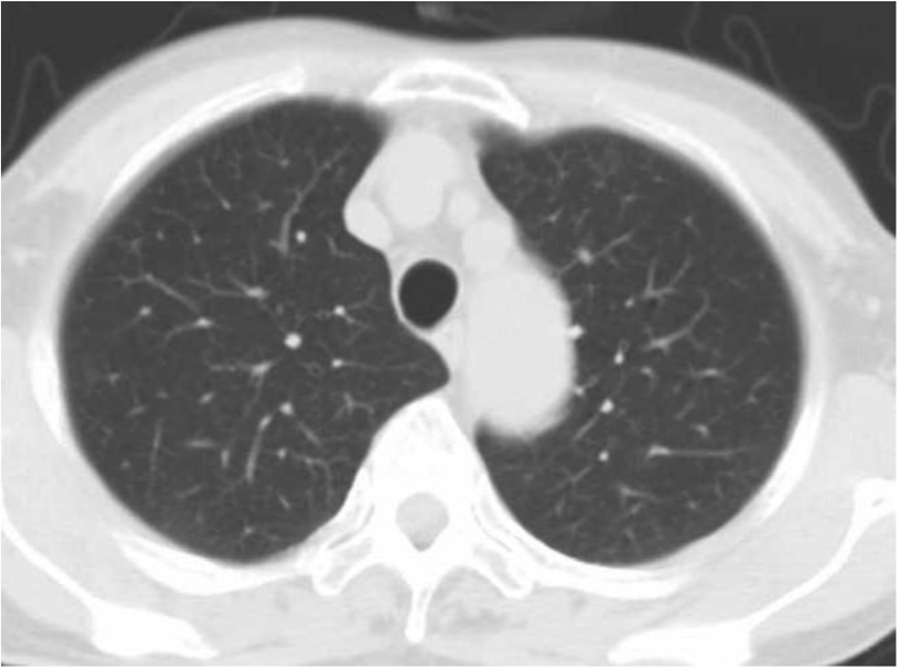
**Chest computed tomography after prednisone treatment of 20mg/day.** Chest computed tomography showing improvement of patchy ground-glass opacities in the bilateral lung.

**Figure 3:**
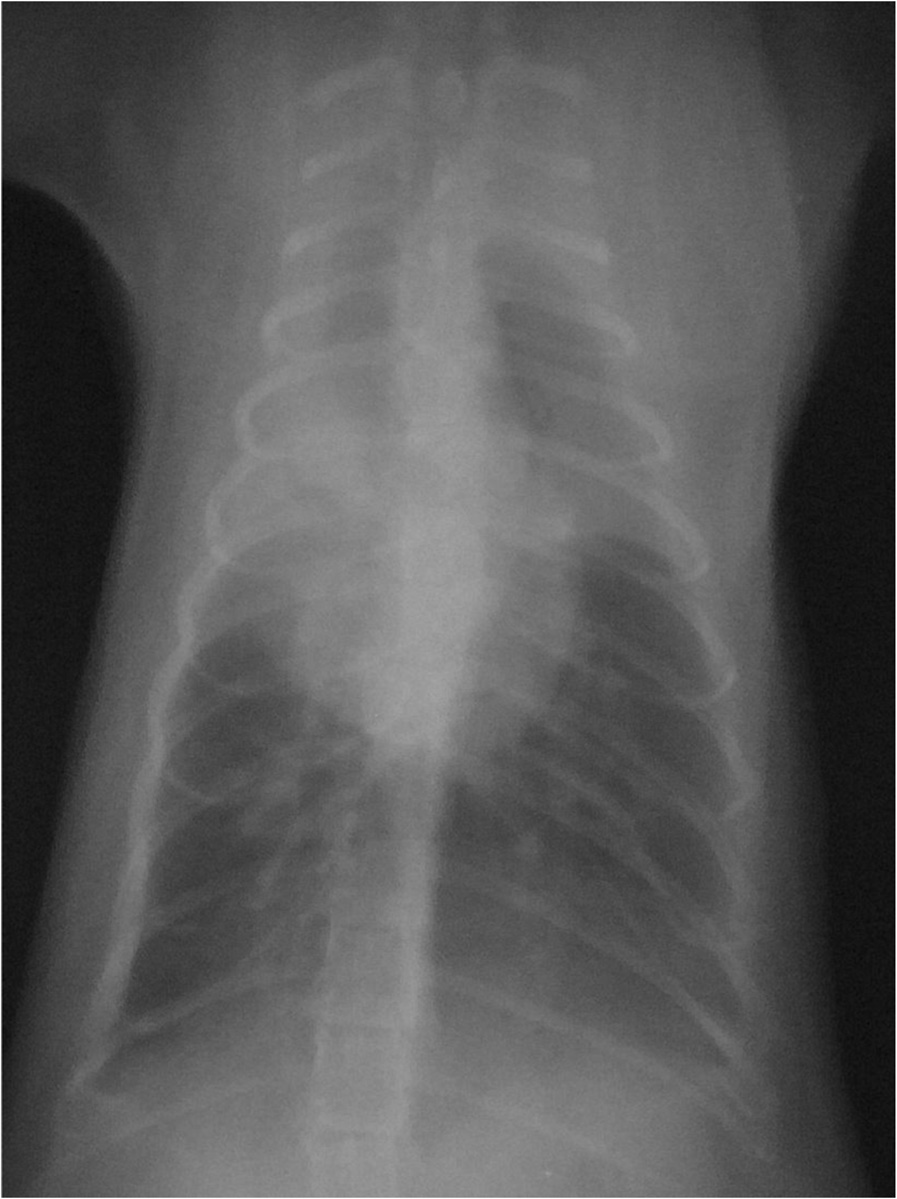
**Chest radiograph of a tame cat before prednisone treatment of 3mg/day.** A chest radiograph of a tame cat revealing ground-glass opacity in the bilateral upper lung fields.

A 66-year-old Japanese non-cigarette smoking woman was given a diagnosis of CEP for 8 years after moving out of her home. She was treated with prednisone; however, recurrence often occurred when prednisone was tapered. In August 2010, she developed respiratory symptoms again with tapering of prednisone (10mg/day). She did not have a history of alcohol abuse, drug abuse, occupational exposure, or exposure to birds. She was afebrile with a respiratory rate of 28 breaths per minute, and complained of non-productive cough and malaise. Auscultation of her chest revealed expiratory wheezes in both lungs, and she had a history of bronchial asthma for 1 year. Results of laboratory findings are as follows: an arterial blood gas analysis while breathing room air showed evidence of respiratory alkalosis (pH7.55; partial pressure of oxygen in arterial blood, 87.7mmHg; partial pressure of carbon dioxide in arterial blood, 30.3mmHg; and bicarbonate, 30.3mEq/L). Her leukocyte count was 7200/uL; 4% were eosinophils. Results of routine serum chemical studies were almost normal except for serum C-reactive protein of 1.1mg/mL (normal, <0.3mg/mL). Antineutrophil cytoplasmic antibodies were absent. Her serum immunoglobulin E (IgE) level was 56.4IU/mL (normal, <173IU/mL). Specific IgE to house dust including *Dermatophagoides pteronyssinus* or *Dermatophagoides farinae*, house dust mites in Japan, and *Aspergillus fumigatus* were all negative (radioallergosorbent tests; class range 0). Her urine analysis was normal, with no active sediments. Stool examinations for ova or parasites were negative. A pulmonary function test demonstrated an obstructive pattern with reduced diffusing capacity of her lungs for carbon monoxide (DL_CO_) (forced vital capacity, 106%; forced expiratory volume_1_, 64%; DL_CO_, 66%). A chest radiograph revealed patchy shadows in her bilateral upper lung fields (Figure 1A). A chest computed tomography showed bilateral patchy ground-glass opacities with subpleural predominance (Figure 1B). Bronchoalveolar lavage fluid recovered 980,000 cells per mL with a differential 62% of eosinophils. Based on negative findings for other causes of eosinophilia, the diagnosis of the recurrence of CEP was established. She was treated with prednisone, 20mg/day, which demonstrated rapid improvement of her clinical picture and chest imaging (Figure 2).Around that same time, her tame cat of 10 years, developed oral breathing, tachypnea (40 breaths/minute) and peripheral eosinophilia (12%). Chest radiography of the tame cat revealed ground-glass opacity in its bilateral upper lung fields (Figure 3). The tame cat, which had lived in the same house as the patient for 8 years, was also diagnosed as having eosinophilic pneumonia and was treated with 3mg/day (1mg/kg) of prednisone for 3 days, resulting in a rapid improvement of the clinical picture. Since eosinophilic pneumonia was diagnosed simultaneously in the patient and the tame cat, it can be suggested that inhaled environmental antigens in the home caused the eosinophilic pneumonia. After moving out of her home, she had no recurrence of CEP with tapering of prednisone. The tame cat also had no recurrence of eosinophilic pneumonia without prednisone.

## Discussion

Because fewer than 10% of patients with CEP improve spontaneously, corticosteroid therapy should be indicated. Most patients undergo prolonged corticosteroid therapy due to common recurrence after cessation or tapering of corticosteroid therapy.

Concerning the etiology, the presence of asthma or the history of atopy accompanies or precedes CEP in over 50% of cases, suggesting that CEP is a chronic hypersensitive reaction related to allergic disorders [1]. CEP occurs predominantly in middle-aged women and non-cigarette smokers, which leads to the speculation that environmental antigens, particularly in the home, contribute to the etiology of CEP. Environmental inhaled antigens often induce acute eosinophilic pneumonia without recurrence of disease because it is easy to detect and avoid antigens such as cigarette smoke [2,3], dust [4], or spiders [5]. However, in cases of eosinophilic pneumonia induced by environmental antigens in the home, recurrence is likely to occur with the tapering of corticosteroid if patients do not move out of their home. In the present case, the inhalation of environmental antigens present in the home might have caused the CEP, based on the negative findings of other causes of pulmonary eosinophilic syndrome and the absence of recurrence after she moved to a different home.

Cats also develop eosinophilic pneumonia through inhaled environmental antigens [6]. Eosinophilic pneumonia recurrence is common in cats, particularly tame ones, since they cannot escape from antigens in the home. In the present case, the tame cat developed eosinophilic pneumonia for the first time while living in the patient’s home, but no recurrences as a result of antigen escape have occurred since the patient and her cat moved to a different home. Environmental antigens in the home can be considered in the etiology of CEP for not only the patient but also the cat, although it is speculative due to the unidentified antigens and no provocative challenge.

## Conclusions

Although CEP is an idiopathic disorder of unknown etiology, our case suggests that inhaled environmental antigens in the home may be associated with the causes of CEP, and that a pet’s disease may give us an important clue for the therapeutic approach of the owner’s disease.

## Consent

The authors obtained written informed consent from the patient to publish this case report and accompanying images. A copy of the written consent is available for review by the Editor-in-Chief of this journal.

## Abbreviations

CEP: Chronic eosinophilic pneumonia; DL_CO_: diffusing capacity of the lung for carbon monoxide; IgE: immunoglobulin E.

## Competing interests

The authors declare that there are no competing interests regarding the publication of this case report.

## Authors’ contributions

TT and MK drafted the manuscript and performed the literature search. JT provided guidance for drafting the manuscript. RK and ET participated in its design and coordination. All authors read and approved the final manuscript.
